# Emerging Insights on Surgical Techniques and Biomaterials for Total Hip and Knee Arthroplasty

**DOI:** 10.1155/2016/1496529

**Published:** 2016-01-27

**Authors:** Kengo Yamamoto, Michiaki Takagi, Hiroshi Ito

**Affiliations:** ^1^Department of Orthopedic Surgery, Tokyo Medical University, 6-7-1 Nishishinjuku, Shinjuku-ku, Tokyo 160-0023, Japan; ^2^Department of Orthopaedic Surgery, Yamagata University Faculty of Medicine, 2-2-2 Iida-Nishi, Yamagata 990-9585, Japan; ^3^Department of Orthopaedic Surgery, Asahikawa Medical University, Midorigaoka Higashi 2-1-1-1, Asahikawa 078-8510, Japan

There has been a great interest by global orthopedic community in the development of new surgical techniques and biomaterials to improve clinical outcomes after total joint hip and knee arthroplasty (THA and TKA). There are currently enormous variations of the surgical treatment options by considering technical factors (surgical approaches, cement/cementless fixation, cup/stem positioning, and a computer-assisted navigation system) as well as prosthetic factors (material structures, properties, and design concepts). In the last decade, the prevalence of osteolysis decreased significantly in THA particularly after the refinement of hip implants including the development of highly cross-linked ultra-high molecular weight polyethylene (HXLPE) and porous coating for cementless fixation. Nevertheless, the postoperative complications such as aseptic loosening, dislocation, and infection still represent the most critical problems limiting the long-term success of the joint replacement surgery. The aim of this special issue is to provide novel concepts beneath the modern joint replacement surgery and to ultimately build successful future operative strategies based on the cutting-edge biomaterials science and clinical experience.

Regarding THA and TKA, this issue presents 13 articles consisting of 9 basic researches, 3 clinical studies, and 1 review paper. The review of R. P. van Hove et al. summarizes the available clinical literatures regarding the surface coating technology of titanium-nitride (TiN) ceramic in orthopaedic implants. K. Watanabe et al. experimentally demonstrated the efficacy of surface modification technology, poly(2-methacryloyloxyethyl phosphorylcholine [MPC])- (PMPC-) grafting, in further reducing HXLPE wear. On the other hand, in molecular scale, Y. Takahashi et al. offered new microstructural insights into the further improvement of wear resistance in HXLPE hip and knee prostheses by means of a novel spectroscopic approach using confocal polarized Raman microprobe spectroscopy. S. Gabarre et al. proposed polycarbonate-urethane as a favorable alternative to traditional bearing surfaces in THA because of its biomechanical characteristics similar to those of joint cartilage. The retrieval study performed by E. K. Fredette et al. contributed to improving our understanding of metal transfer on CoCr and ceramic femoral head surfaces, which may lead to more accurate HXLPE wear studies through more realistic recreation of metal transfer in* in vitro* wear simulation testing.

Excellent short to midterm clinical outcomes were presented by A. Hozumi et al. in anatomically difficult Asian patients with developmental dysplasia of hips, postosteotomy hip, and posterior pelvic tilt using the S-ROM-A prosthesis. The study of P. Sa-ngasoongsong et al. showed that, in order to minimize blood loss without significant increase in systemic absorption, low-dose intra-articular tranexamic acid (IA-TXA) application in TKA with prolonged clamping drain method was a safe and effective blood conservative technique. In preventing and treating postoperative infection, T. Kabata et al. showed the great success of short-term clinical trial using iodine-supported THA implants, which they have originally developed in the recent years. For the detection and diagnosis of occult infection, J. T. Kempthorne et al. recommended conventional routine intraoperative sampling techniques rather than ultrasound sonication of the removed prosthesis potentially leading to the increased risk of contamination.

From the viewpoints of preventive medicine against hip fracture and knee osteoarthritis (OA), the risk assessments were conducted in the two studies. According to finite element models constructed from quantitative computed tomography (QCT), it was found by H. Kheirollahi and Y. Luo that femoral neck and the intertrochanteric region had a greater risk of fracture than other parts of the femur, and women were more prone to hip fracture than men. On the other hand, N. Lanocha-Arendarczyk et al. evaluated the concentrations of 10 chemical elements (F^−^, K, Zn, Fe, Sr, Pb, Mn, Se, Cd, and THg) in tibial bone with OA and found statistically significant effects of environmental exposure factors including smoking, seafood diet, and geographical distribution on the levels of the elements.

In the technical aspects of THA including operational planning, T. A. Weber et al. reported improved hip joint reaction forces and patient's gait parameters by their originally developed method of a novel computer-assisted operation for minimal-invasive THA following the concept of “femur first/combined anteversion,” which incorporates various aspects of performing a functional optimization of the stem and cup position (CAS FF), as compared to the conventional THA procedures. On the other hand, for TKA, the optimum design of femoral cutting guide was suggested by O.-R. Kwon et al. as patient-specific instruments (PSI), which can potentially shorten operative time and improve implant alignment.

We hope that the knowledge reported in this issue will be a hint to promote new study for the readers working in the same field and will lead to future development of new surgical techniques and implant devices that can respond to the increasing expectations of patients.

